# Sensory-based visual attention in autism: from normalization to adaptive support

**DOI:** 10.3389/fpsyt.2026.1756363

**Published:** 2026-01-28

**Authors:** Paola Chilà, Antonino Pricipato, Gaia Roccaforte, Flavio Corpina, Chiara Marraffa, Gaetano Vivona, Chiara Failla, Giovanni Pioggia, Flavia Marino

**Affiliations:** 1 Institute for Biomedical Research and Innovation (IRIB), National Research Council of Italy (CNR), Messina, Italy; 2 Faculty of Psychology, International Telematic University Uninettuno, Roma, Italy; 3 Digital Agenda and Processes Office, Directorate General, National Research Council of Italy (CNR), Rome, Italy; 4 Department of Biomedical, Dental and Morphological and Functional Imaging Sciences, University of Messina, Messina, Italy; 5 Multi-Specialist Clinical Institute for Orthopaedic Trauma Center (COT), Messina, Italy; 6 Department of Cognitive, Psychological Science and Cultural Studies, University of Messina, Messina, Italy; 7 Department of Mental Health, Azienda Sanitaria Provinciale (ASP) Trapani, Cittadella della Salute, Trapani, Italy

**Keywords:** adaptive support, autism spectrum disorder, technologies, visual attention, well-being

## Abstract

Over the years, attentional characteristics such as reduced attention to faces or eye contact have been considered impairments and in need of correction in people with autism. This work proposes a reinterpretation of visual attention in people with autism, going beyond the traditional deficit-based approach. It proposes a shift in perspective by examining the characteristics of visual attention in people with autism as a resource for personal orientation strategies in complex and challenging environments. To better study and observe these mechanisms, the use of digital technologies such as mobile eye trackers, virtual reality, and other digital technologies can offer valuable support for better delineating the different strengths of this type of attentional modality, which deviates from the “norm.” Therefore, rather than focusing on correction, the shift in perspective should focus much more on the understanding, well-being, and autonomy of people with autism. Viewing autistic attention not as a problem, but as a resource to be valued, is the first step towards building a truly inclusive society.

## Introduction

Visual attention can be considered as a selective filter that allows us to identify which stimuli to perceive, process and integrate in an environmental context to then be integrated into higher cognitive processes (1). Some authors have hypothesized the existence of an innate “social mind” since in typical developmental paths emerge attentional preferences and a spontaneous propensity towards salient social stimuli such as human faces (2). This early orientation towards the social is considered a crucial basis for language acquisition, emotional regulation and interpersonal understanding. For many years, atypical development has been attributed to a deviation from these early social attention patterns. In autistic people, a large body of studies has documented the emergence of attentional preferences often oriented towards non-social elements, peripheral details or specific perceptual aspects, rather than towards complex social scenes (3–6). These differences have been framed within models that attribute a reduced social motivation typical of people with autism (7) or to the inability to attribute mental states to others (8). New lines of research instead suggest that these attentional preferences may represent functional and adaptive strategies, shaped by peculiar neurological and perceptual configurations (9, 10). In this perspective, reduced attention to the face does not necessarily imply social disinterest, but may reflect a different way of processing information, based on criteria of predictability, structure and optimal sensory load (11). This perspective invites us to move beyond the idea of ​​a “normative” development as a univocal reference, promoting a more nuanced understanding of attentional differences as valid expressions of alternative cognitive architectures. In this context, digital technologies take on a central role as tools for ecological observation and personalized support. Technologies such as mobile eye-trackers, adaptive interfaces and immersive sensory environments allow not only to detect attention patterns in natural situations, but also to build tailor-made experiences that enhance individual resources and interests, promoting authentic involvement without imposing normative objectives (12–14). Introducing these tools right from the evaluation phase means not only improving the accuracy of clinical observation, but also redefining the intervention criteria, placing the emphasis not on correcting behavior, but on building paths that respect neurodivergent functioning, promoting well-being, autonomy and meaningful participation.

## Early divergence in attentional priorities

Longitudinal studies conducted with eye-tracking on infants later diagnosed with autistic neurodivergence detect a gradual decline in eye fixation times as early as 2 to 6 months of age (15), so divergent patterns of visual attention may be detectable even before formal diagnosis. Moreover, such atypical patterns have been mainly attributed to social stimuli such as faces and gestures as opposed to high-contrast geometric patterns and visually predictable stimuli (16, 17). Studies on neurodevelopmental condition, particularly autism spectrum disorder (ASD), confirm the precociousness of attentional deficits (18), which explains the impaired interaction with social stimuli and consequently the development of social cognition and corresponding networks. Difficulties are found in processing social cues such as emotional expression (19–21) and gaze (22–24), as well as in abstracting invariant features necessary for facial recognition (25–27), in fact, in experimental settings, children and adults with ASD often pay attention to faces while not extracting the same crucial information from faces as individuals with typical development (18). For many autistic people divergence, social stimuli represent unpredictable or overstimulating stimuli therefore they are inclined to develop a natural tendency to favor more stable and structured visual elements in their surroundings. Can this tendency of theirs affect their development, are people with autistic divergence less socially engaged because they pay attention differently or do they pay attention differently because stimuli, especially social stimuli, are less rewarding or more cognitively challenging?

## Beyond the deficit model

The dominant view of atypical visual attention in autistic people is based on the concept of deficit because it is believed that decreased attention to social stimuli reflects a lack of interest, impaired social cognition, or a delay in the development of shared intentionality. Therefore, joint attention or gaze training has been developed, although this could risk simplifying a complex phenomenon or ignoring the inner logic of autistic cognition. A more nuanced interpretation, however, recognizes that attentional differences may reflect a valid rather than pathological perceptual style. For example, increased attention to more static objects or patterns could be a compensatory strategy that facilitates predictability and reduces cognitive load in sensorially overloaded or socially ambiguous environments (28). Reframing the concept could therefore have different implications for both research and clinical practice, as if interventions that aim to “normalize” visual behavior might not consider the underlying sensory and emotional costs. A strengths-based approach, on the other hand, should understand the individual attentional profile and build intervention strategies based on it. Reframing visual attention in autism as divergent rather than deficit opens new avenues for both scientific inquiry and clinical innovation. This approach involves an appreciation of neurodiversity and an understanding of attentional differences as such, not just as precursors to pathology, but as alternative, coherent, and meaningful ways of interacting with the environment (29, 30), although specific enhancement should not be lacking. This involves shifting attention toward a neurodiversity-centered rather than pathologizing view, so that perceptual atypicality is studied for what it is, and not just for what it lacks relative to an assumed norm (31). Interventions that aim only at correcting rather than capturing the strengths of people with autism are shown, based on what has been analyzed, to be less effective than those that make use of visual and/or textual supports and personal interests (32). Thus, it is important to recognize and understand attention differences so that they are not perceived as obstacles, but specific aspects on which to set intervention. To do this, clinical practice and research are today called upon to equip themselves with tools capable of detecting and enhancing these perceptive modalities in their real context: digital technologies offer, in this sense, new opportunities.

## Digital technologies to enhance individual attention strategies

Within a clinical perspective oriented towards neurodiversity, digital technologies represent promising tools not to normalize, but to understand and support the spontaneous attentional modalities of autistic people. In particular, the use of technologies such as portable eye-trackers, immersive sensory environments, adaptive interfaces and interactive applications allows us to observe and support attention in an ecological, respectful and personalized way. For example, the use of mobile eye-tracking in natural contexts (such as home or school) allows to detect spontaneous attentional patterns in a non-invasive way, offering valuable data on where and how attention is distributed without forcing the person to rigid or potentially stressful tasks. This information can be used to build individual attentional profiles, useful for designing targeted interventions that integrate visual interests, preferred sensory channels and the need for predictability of stimuli. In parallel, the use of adaptive educational apps or digital interfaces capable of modulating content based on detected attention patterns can enhance learning and communication starting from the specific perceptive functioning of the user. In this way, attention is not forced towards normative objectives (e.g. eye contact), but accompanied in the direction of individual interests and resources. Studies such as that of Powell et al. (33) have shown that training with gaze-contingent eye-tracking improves sustained attention without forcing the gaze towards the face, but adapting to the cognitive functioning mode of each child. Even in the therapeutic field, controlled multisensory environments – such as Snoezelen rooms or immersive platforms – allow for the safe testing of different stimulus-response configurations, observing how attention varies in relation to light intensity, auditory stimuli, movement or the presence of simulated social elements. This dynamic evaluation supported by technology allows for the identification of optimal activation and regulation conditions, paving the way for truly personalized interventions. The integration of digital technologies into clinical practice can profoundly transform the way in which we observe and work with autistic attention: from an element to be corrected to a resource to be understood, supported and valorized. This change of perspective also implies a review of clinical objectives, efficacy criteria and the role of technology itself. The following table synthetically compares the traditional approach, centered on normalization, with the one informed by neurodiversity, which aims to valorize individual profiles (See **Table 1**):

**Table 1:** Traditional vs. neurodiversity-informed approaches.

Characteristic	Traditional/normative approach	Neurodiversity-informed/adaptive approach
Main objective	Normalize attention toward social stimuli (e.g., face, eyes)	Understand and enhance spontaneous attention patterns
Definition of success	Increase in eye contact, imitation of neurotypical behaviors	Improvement in communication, emotional regulation, and personal well-being
Role of technology	Tool to shape or reinforce target behaviors (e.g., digital ABA)	Tool to observe, adapt, and create personalized experiences
Example of technological use	Training to fix gaze on eyes	Eye-tracking that adapts content to attentional style
Type of stimuli used	Standardized, often social stimuli (e.g., human face looking at the user)	Stimuli based on personal interests, predictable and sensorially regulated
Clinical implications	Increase observable behaviors for “social integration”	Support effective communication and regulation based on the individual profile
Main challenges	Risk of stress, masking, disregard for internal functioning	Requires more time, fine observation, and specific training

## Discussion

The characteristics of visual attention in autism must be read as an adaptive and consistent expression of divergent perceptual modalities to be in line with an innovative perspective of research and clinical intervention. The attentional differences observed in the early stages of development are not necessarily indicative of social disinterest or impairment, but may reflect spontaneous strategies to manage sensory complexity, cognitive load and the unpredictability of social stimuli (28, 31). In the digital era, new technologies can play an important role in favoring the transition towards a new understanding in supporting and enhancing attentional skills in autism by moving from being tools aimed at normalizing behavior (e.g., increasing eye contact) to means to observe, understand and improve individual attentional trajectories (34). This allows for the construction of personalized interventions that do not impose a normative model, but that adapt to the sensory and motivational profile of the person (32, 35). From a clinical point of view, it means moving from interventions focused on the “correction” of attention (such as gaze training) to practices that enhance its communicative and regulatory functionality. Using visual supports, predictable environments, stimuli related to interests and an experiential design sensitive to sensory load not only increases effectiveness, but reduces the risk of stress, masking and failure to recognize individual identity (10). The conscious integration of technology paves the way for dynamic assessment and therapy models, which are not limited to measuring static behaviors, but follow the evolution of attention over time and contexts, offering tools to build authentic and sustainable therapeutic and educational relationships. Adopting a perspective centered on neurodiversity, supported by technologies that respect individual functioning, therefore means redefining the criteria of clinical efficacy, placing at the center not forced adaptation, but autonomy, well-being and the valorization of the perceptive and cognitive resources of each person.

## Data Availability

The original contributions presented in the study are included in the article/Supplementary Material. Further inquiries can be directed to the corresponding author.

## References

[1] Posner MI Rothbart MK . Educating the human brain. Washington, DC: American Psychological Association (2007). doi: 10.1037/11519-000

[2] Wagner JB Luyster R Yim H Tager-Flusberg H Nelson CA . The role of early social attention in the development of social communication. Dev Sci. (2013) 16:284–98. doi: 10.1111/desc.12026, PMID: 23587039

[3] Klin A Jones W Schultz R Volkmar F Cohen D . Visual fixation patterns during viewing of naturalistic social situations as predictors of social competence in individuals with autism. Arch Gen Psychiatry. (2002) 59:809–16. doi: 10.1001/archpsyc.59.9.809, PMID: 12215080

[4] Akin-Bulbul I Ozdemir S . Evaluation of the social attention hypothesis: do children with autism prefer to see objects rather than people? J Autism Dev Disord. (2024), 1–15. doi: 10.1007/s10803-024-06596-9, PMID: 39546170

[5] Apicella F Costanzo V Purpura G . Are early visual behavior impairments involved in the onset of autism spectrum disorders? Insights for early diagnosis and intervention. Eur J Pediatr. (2020) 179:225–34. doi: 10.1007/s00431-019-03562-x, PMID: 31901981

[6] Chen J Wei Z Xu C Peng Z Yang J Wan G . Social visual preference mediates the effect of cortical thickness on symptom severity in children with autism spectrum disorder. Front Psychiatry. (2023) 14:1132284. doi: 10.3389/fpsyt.2023.1132284, PMID: 37398604 PMC10311909

[7] Chevallier C Kohls G Troiani V Brodkin ES Schultz RT . The social motivation theory of autism. Trends Cogn Sci. (2012) 16:231–9. doi: 10.1016/j.tics.2012.02.007, PMID: 22425667 PMC3329932

[8] Baron-Cohen S . Mindblindness: An essay on autism and theory of mind. Cambridge, MA: MIT Press (1995).

[9] Robertson CE Baron-Cohen S . Sensory perception in autism. Nat Rev Neurosci. (2017) 18:671–84. doi: 10.1038/nrn.2017.112, PMID: 28951611

[10] Mottron L . A radical change in our autism research strategy is needed: Back to prototypes. Autism Res. (2021) 14:2213–20. doi: 10.1002/aur.2494, PMID: 34077611

[11] Del Bianco T Mason L Charman T Tillman J Loth E Hayward H . Temporal profiles of social attention are different across development in autistic and neurotypical people. Biol Psychiatry: Cogn Neurosci Neuroimaging. (2021) 6:813–24. doi: 10.1016/j.bpsc.2020.09.004, PMID: 33191160

[12] Fletcher-Watson S Hampton S . The potential of eye-tracking as a sensitive measure of behavioural change in response to intervention. Sci Rep. (2018) 8:14715. doi: 10.1038/s41598-018-32444-9, PMID: 30279422 PMC6168486

[13] Jeong J Kim SH Yang HJ Lee GA Kim S . GazeHand: A gaze-driven virtual hand interface. IEEE Access. (2023) 11:133703–16. doi: 10.1109/ACCESS.2023.3337372

[14] Jeong D Jeong M Yang U Han K . Eyes on me: Investigating the role and influence of eye-tracking data on user modeling in virtual reality. PloS One. (2022) 17:e0278970. doi: 10.1371/journal.pone.0278970, PMID: 36580442 PMC9799296

[15] Jones W Klin A . Attention to eyes is present but in decline in 2–6-month-old infants later diagnosed with autism. Nature. (2013) 504:427–31. doi: 10.1038/nature12715, PMID: 24196715 PMC4035120

[16] Bast N Mason L Ecker C Baumeister S Banaschewski T Jones EJ . Sensory salience processing moderates attenuated gazes on faces in autism spectrum disorder: a case–control study. Mol Autism. (2023) 14:5. doi: 10.1186/s13229-023-00537-6, PMID: 36759875 PMC9912590

[17] Kojovic N Cekic S Castañón SH Franchini M Sperdin HF Sandini C . Unraveling the developmental dynamic of visual exploration of social interactions in autism. Elife. (2024) 13:e85623. doi: 10.7554/eLife.85623, PMID: 38192197 PMC10876216

[18] Chawarska K Shic F . Looking but not seeing: Atypical visual scanning and recognition of faces in 2 and 4-year-old children with autism spectrum disorder. J Autism Dev Disord. (2009) 39:1663–72. doi: 10.1007/s10803-009-0803-7, PMID: 19590943 PMC4878114

[19] Golan O Baron-Cohen S Hill JJ Golan Y . The ‘Reading the Mind in Films’ task: Complex emotion recognition in adults with and without autism spectrum conditions. Soc Neurosci. (2006) 1:111–23. doi: 10.1080/17470910600980986, PMID: 18633780

[20] Hobson RP Ouston J Lee A . Emotion recognition in autism: Coordinating faces and voices. psychol Med. (1988) 18:911–23. doi: 10.1017/S0033291700009843, PMID: 3270834

[21] Kätsyri J Saalasti S Tiippana K von Wendt L Sams M . Impaired recognition of facial emotions from low-spatial frequencies in Asperger syndrome. Neuropsychologia. (2008) 46:1888–97. doi: 10.1016/j.neuropsychologia.2008.01.005, PMID: 18314147

[22] Dalton KM Nacewicz BM Johnstone T Schaefer HS Gernsbacher MA Goldsmith HH . Gaze fixation and the neural circuitry of face processing in autism. Nat Neurosci. (2005) 8:519–26. doi: 10.1038/nn1421, PMID: 15750588 PMC4337787

[23] Joseph RM Tanaka J . Holistic and part-based face recognition in children with autism. J Child Psychol Psychiatry. (2003) 44:529–42. doi: 10.1111/1469-7610.00142, PMID: 12751845

[24] Senju A Yaguchi K Tojo Y Hasegawa T . Eye contact does not facilitate detection in children with autism. Cognition. (2003) 89:B43–51. doi: 10.1016/S0010-0277(03)00058-9 12893128

[25] Annaz D Karmiloff-Smith A Johnson MH Thomas MS . A cross-syndrome study of the development of holistic face recognition in children with autism, Down syndrome, and Williams syndrome. J Exp Child Psychol. (2009) 102:456–86. doi: 10.1016/j.jecp.2008.11.009, PMID: 19193384

[26] Boucher J Lewis V . Unfamiliar face recognition in relatively able autistic children. J Child Psychol Psychiatry. (1992) 33:843–59. doi: 10.1111/j.1469-7610.1992.tb01963.x, PMID: 1634592

[27] Klin A Sparrow SS de Bildt A Cicchetti DV Cohen DJ Volkmar FR . A normed study of face recognition in autism and related disorders. J Autism Dev Disord. (1999) 29:499–508. doi: 10.1023/A:1022150913500, PMID: 10638462

[28] Haskins AJ Mentch J Botch TL Garcia BD Burrows AL Robertson CE . Reduced social attention in autism is magnified by perceptual load in naturalistic environments. Autism Res. (2022) 15:2310–23. doi: 10.1002/aur.2829, PMID: 36207799 PMC10092155

[29] Klin A Lin DJ Gorrindo P Ramsay G Jones W . Two-year-olds with autism orient to non-social contingencies rather than biological motion. Nature. (2009) 459:257–61. doi: 10.1038/nature07868, PMID: 19329996 PMC2758571

[30] Bedford R Pickles A Gliga T Elsabbagh M Charman T Johnson MH . Additive effects of social and non-social attention during infancy relate to later autism spectrum disorder. Dev Sci. (2014) 17:612–20. doi: 10.1111/desc.12139, PMID: 25089324 PMC4253134

[31] Pellicano E Burr D . When the world becomes ‘too real’: A Bayesian explanation of autistic perception. Trends Cogn Sci. (2012) 16:504–10. doi: 10.1016/j.tics.2012.08.009, PMID: 22959875

[32] Jaswal VK Akhtar N . Being versus appearing socially uninterested: Challenging assumptions about social motivation in autism. Behav Brain Sci. (2019) 42:e82. doi: 10.1017/S0140525X18001826, PMID: 29914590

[33] Powell G Wass SV Erichsen JT Leekam SR . First evidence of the feasibility of gaze-contingent attention training for school children with autism. Autism. (2016) 20:927–37. doi: 10.1177/1362361315617880, PMID: 26862085 PMC5070492

[34] Fletcher-Watson S Happé F Bird G . Autism and empathy: What are the real links? Autism. (2020) 24:515–7. doi: 10.1177/1362361320901627, PMID: 31674189

[35] Lyu Y An P Xiao Y Zhang Z Zhang H Katsuragawa K . Eggly: Designing mobile augmented reality neurofeedback training games for children with autism spectrum disorder. Proc ACM Interact Mob Wearable Ubiquitous Technol. (2023) 7:1–29. doi: 10.1145/3596251

